# Synergistic Antioxidant Activity of Four—Component Mixture of Essential Oils: Basil, Cedarwood, Citronella and Thyme for the Use as Medicinal and Food Ingredient

**DOI:** 10.3390/antiox12030577

**Published:** 2023-02-25

**Authors:** Tomasz Baj, Grażyna Kowalska, Radosław Kowalski, Jolanta Szymańska, Guoyin Kai, Henrique Douglas Melo Coutinho, Elwira Sieniawska

**Affiliations:** 1 Department of Pharmacognosy with Medicinal Plants Garden, Medical University of Lublin, 1 Chodźki Str., 20-093 Lublin, Poland; 2 Department of Tourism and Recreation, University of Life Sciences in Lublin, 15 Akademicka Str., 20-950 Lublin, Poland; 3 Department of Analysis and Food Quality Assessment, University of Life Sciences in Lublin, 8 Skromna Str., 20-704 Lublin, Poland; 4 Department of Integrated Paediatric and Adult Dentistry, Medical University of Lublin, 6 Chodźki Str., 20-093 Lublin, Poland; 5 Zhejiang Provincial International S&T Cooperation Base for Active Ingredients of Medicinal and Edible Plants and Health, Jinhua Academy, School of Pharmaceutical Sciences, Zhejiang Chinese Medical University, Hangzhou 310053, China; 6 Departamento de Química Biológica, Universidade Regional do Cariri, Rua Cel. Antônio Luíz-Pimenta, Crato 63105-110, CE, Brazil; 7 Department of Natural Products Chemistry, Medical University of Lublin, 1 Chodźki Str., 20-093 Lublin, Poland

**Keywords:** essential oils, simplex-lattice mixture design, statistical modelling, DPPH, food preservation medicinal applications

## Abstract

Mixture design is a statistical tool used to obtain the maximum desired effect using the minimum number of experiments. The aim of the presented work was the optimization of the composition of a mixture of essential oils from basil, citronella, cedarwood and thyme using simplex-lattice mixture design method. The optimized parameter was an antioxidant activity measured in DPPH assay and expressed as effective concentration (EC_50_). The test results showed an interesting synergy between the components of essential oils. The prepared binary and quaternary mixtures were characterized by higher activity than simple average activity. The designed mixture with approximated highest antioxidant activity was composed of: 54.4% citronella essential oil, 33.0% thyme essential oil, 9.2% cedarwood essential oil and 3.4% basil essential oil and its approximated activity was in agreement with experimental values. This work confirmed that it is possible to approximate the best antioxidant composition of four essential oils used as a potential medicinal and food ingredient.

## 1. Introduction

Physico-chemical factors and microbial degradation are well-known reasons for food spoilage. Chemical factors causing food deterioration include enzymatic and non-enzymatic reactions, light exposure or rancidity and are mainly due to oxidation [1]. The degradation products and microbial metabolites are the cause of unpleasant flavors and changes in texture of food resulting in decreased organoleptic characteristics, safety and nutritional value [2]. Aside from microbial growth, oxidative deterioration is the major cause of food spoilage. Oxidation in foods leads to the formation of reactive oxygen species in both the aqueous and lipid, phases and progression of oxidation takes place after free radical formation by electron transfer and hydrogen atom transfer. Protein and lipid oxidation occurs simultaneously in meat and fish products. This results in a lowering of its nutritional value and shelf life and causes the formation of potentially toxic substances [1,2]. In contrast, oxidation in fruits and vegetables is manifested mainly through darkening and softening, and ends in alteration of vitamins content [2]. Transition metals present in food may additionally initiate oxidation by direct interaction with oxygen and by generation of superoxides, perhydroxides and hydroxyl radicals which can then bring about further oxidation, co-oxidation and propagation by transition metal catalysers [1].

Because of the increasing demand for healthy and unprocessed food, many products containing polyunsaturated fatty acids are packed in oxygen-permeable wrappings, thus they are highly susceptible to oxidative deterioration [1]. The natural consequence of this situation is a need for the presence of natural and safe antioxidants in food-items. Since spices are well-known food additives used since ancient times to improve flavours and to prolong the storage stability of foods, they can be considered as food preservatives. Spices are rich in volatiles, whereas essential oils are volatiles obtained from aromatic plants. Essential oils are abundant in phenolic compounds and are characterized by antioxidant and antimicrobial properties [3]. The antioxidant activities of phenolics present in essential oils are due to free radical-scavenging via hydrogen-donation, singlet oxygen quenching or metal ion chelation [1]. The antioxidant and antimicrobial activity of essential oils have been reported in meat, fish, fruits, vegetables and dairy products [2,3,4,5,6], and most essential oils are generally recognized as safe (GRAS) by the US Food and Drug Administration and by European Commissions. More and more research is being conducted on the use of essential oils as natural antioxidants. Anthony et al. [7] studied the antioxidant activity of 423 essential oils from 48 different botanical families. In the test with the DPPH reagent, 73 of them were found to have an activity of 50% or more. The most active turned out to be essential oils obtained from plants of the Lamiaceae and Myrtaceae families. In the essential oils with high antioxidant activity, the main components were phenolic terpenes (thymol, carvacrol and eugenol). Components such as 1,8-cineole, linalool, borneol and terpinen-4-ol and sesquiterpenes [7] were also able to capture free radicals. Being mixtures of many chemical constituents, essential oils are usually tested separately as food preservatives [2,8,9,10]. However, their activity can be potentiated when several essential oils are blended. This can bring benefits ranging from lower concentrations needed, to lowered risks of alterations of food flavour and aroma. The combined effects of two and more essential oils has been extensively studied in terms of their antimicrobial action by calculation of the fractional inhibitory concentration index [11,12,13]. This methodology can also be applied for the evaluation of ternary combinations; however, it is not always accurate [14]. The prediction of the composition of the mixture with best activity can be obtained using statistical methods, as described by Eriksson et al. [15]. The Mixture Design methodology enables the correlation of independent variables (proportions of the components under investigation) with dependent variables (otherwise known as response values), which depend only on the proportions of the mixture components [16,17].

The strategy of simplex-centroid design has already been applied for a design of mixture of essential oils with best antimicrobial properties [14,18,19]. However, little is known about the synergistic antioxidant effects of essential oils studied using simplex-lattice design, which is a more complex system as compared to the simplex-centroid design, and provides greater freedom of data interpretation. Up to now, only ternary mixtures of essential oil were analysed for their antioxidant effects [20]. In addition, limited studies have been conducted for a design of the mixture of more than three essential oils. Hence, in this work we attempted to ascertain whether it is possible to approximate the best antioxidant blend of four essential oils using simplex-lattice mixture design and the 2,2-diphenyl-1-picrylhydrazyl (DPPH) test. The chosen essential oils were used as a model system of components with higher and lower antioxidant activity so as to design a most effective blend. All studied essential oils are generally recognized as safe [9] and have been used as flavour ingredients by the food industry [9,21].

## 2. Materials and Methods

### 2.1. Chemicals

2,2-diphenyl-1-picrylhydrazyl (DPPH) was obtained from Sigma-Aldrich, St. Louis, MO, USA, and methanol of analytical grade was obtained from Avantor Performance Materials Poland S.A., Gliwice, Poland. The essential oils used in this study were commercially available: basil essential oil (*Ocimum basilicum*, Aromatika Ukraine LLC, Kive, Ukraine), cedarwood essential oil (*Juniperus virginiana*, Bamer, Włocławek, Poland), citronella essential oil (*Cymbopogon nardus*, Pollena Aroma, Nowy Dwór Mazowiecki, Poland) and thyme essential oil (*Thyme vulgaris*, Avicena-Oil, Wrocław, Poland). Essential oils were purchased from the herbal store.

### 2.2. Mixtures of Essential Oils

The authentication of purchased essential oils was carried out by means of gas chromatography-mass spectrometry (Shimadzu GC-2010 Plus coupled to a Shimadzu QP 2010 Ultra mass spectrometer; Shimadzu, Kyoto, Japan) according to methodology described previously [22]. In undertaking this, 50.0 mg of each essential oil was exactly weighed and dissolved in 1000 µL of methanol, then vortexed. The essential oils were mixed as described in Table 1.

### 2.3. Evaluation of Antioxidant Activity of Mixtures of Essential Oils

The antioxidant activities of mixtures of essential oil as calculated according to the statistical model, were evaluated by means of the addition of DPPH reagent as based on the reaction described by Brand-Williams et al. [23]. The essential oils were investigated in concentrations from 0.1 to 50.0 mg/mL, while the DPPH was dissolved in methanol to obtain a concentration of 78.88 µg/mL. According to procedure, pure essential oils and their blends were added to the wells of a 96-well plate in an aliquot of 50 µL. Subsequently, 150 µL of DPPH solution was added to each well, the plate was then shaken for 10 s. and incubated without access to light for 30 min. After incubation, the absorbance was measured spectrophotometrically at 515 nm (BioTek, ELx808, Winooski, VT, USA). The EC_50_ parameter was determined using the program Gen5 by applying the equation from the four-parameter logistic model (4PL) (BioTek, Winooski, VT, USA, Software ver. 3.08.01). The EC_50_ value was calculated for each studied mixture. Experiments were performed in five replicates and presented as mean. The negative control was a DPPH solution where methanol was added instead of the test solution.

### 2.4. Statistical Analysis

The statistical program Statistica 12^®^ (StatSoft Inc., Tulsa, OK, USA) was used to create the experimental design and to model data analysis. A simplex-lattice design with number of components = 4 and polynominal degree m = 2 was used for the preparation of a four-component mixture of essential oils with an optimal antioxidant value (EC_50_). In the preparation of the designed mixture, 15 different measuring systems were used: 4 being pure essential oils; 6 systems being two-component mixtures; 4 mixtures being quadruple internal systems with a greater percentage share of one component (62.5:12.5:12.5:12.5, respectively); one mixture being a centre point, which contained all of the tested essential oils in equal amounts (Table 1).

## 3. Results and Discussion

The chemical composition of the studied essential oils confirmed their origin (Table S1). The main ingredients of citronellal essential oil were: citronellal (31.2%), geraniol (20.6%) and citronellol (13.4%). Methyl chavicol dominated in basil essential oil (80.6%); thujopsene (23.2%), α−cedrene (17.3%) and cedrol (15.3%) were predominant in cedarwood essential oil; thymol (42.9%) and p-cymene (22.4%) dominated in thyme essential oil. The chemical characteristics of the essential oils were in agreement with previously published data [21,24,25,26].

Antioxidant activity of essential oils selected for this study was described as EC_50_ value and was in a range from 1.08 to 4.04 mg/mL (Table 1). The weakest activity was obtained for cedarwood essential oil, which was then further mixed with essential oil showing higher antiradical power: citronella essential oil, thyme essential oil and basil essential oil, resulting in values of 1.08, 1.47 and 1.36 mg/mL, respectively. The literature data confirmed the antioxidant properties of basil essential oil, indicating that a chemotype rich in methylchavicol is a more potent antioxidant (EC_50_ 0.21 mg/mL with DPPH) than a chemotype rich in eugenol and linalool (EC_50_ 4.04 mg/mL with DPPH) [27]. Additionally, Shirazi and co-workers [28] found that the methylchavicol chemotype of basil essential oil was equally effective in scavenging reactive oxygen species and reactive nitrogen species. *Cymbopogon nardus* essential oil also proved to have antioxidant activity by scavenging more than 80% of all DPPH free radicals [29], and inhibiting lipid peroxidation in cell culture-based systems [30]. However, the most extensively studied essential oil was thyme essential oil. This indicated antioxidant activity values from 0.26 mg/mL to 4.05 mg/mL (EC_50_), with DPPH depending on the chemotype tested [25,31]. The main ingredients of thyme essential oil (thymol and carvacrol) demonstrated the ability to donate hydrogen to free radicals and neutralize them [25]. The antioxidant activities of the described essential oils have already found use in food preservation. Basil essential oil formulated in sausage salami preserves lipids from oxidation during processing and storage [9], while thyme essential oil is known to improve the chemical stability of roasted sunflower seeds by preventing lipid oxidation and the development of rancid flavours [32]. Moreover, essential oil of *C. nardus* and *Ocimum basilicum* were previously found to significantly control anthracnose in banana, and increase banana shelf-life up to 21 days. These effects come about due to their antioxidant and antimicrobial properties [33].

Besides their use as pure essential oils, they can be combined with polylactic acid (PLA) to produce packaging films. Zeid et al. [34] evaluated the antioxidant properties of films containing thyme, rosemary or oregano essential oils. In the DPPH test, the authors showed that rosemary essential oil was characterized by the lowest loss of antioxidant activity (3.6%). Films containing essential oils may extend the shelf life of minced fish in terms of the degree of lipid oxidation. The decrease in the degree of oxidation of minced fish muscle on the 4th day was 5.1% for foil with thyme, rosemary or oregano oil; 20.2% and 47.9%, respectively [34].

The aim of the presented work was the optimization of the composition of a mixture of essential oils from cedar, thyme, basil and citronella using the statistical Mixture Design method. Mixture design is a statistical method employed to obtain the maximum effect while using the minimum number of experiments. In our work, the optimized parameter was the antioxidant activity expressed as EC_50_. The test results showed an interesting synergy between the components of the oils (Table 1). When weak antioxidant (cedarwood essential oil) was mixed with any of the essential oils showing better scavenging properties, the prepared binary and quaternary mixtures were characterized by higher activity than simple average activity. What is more, when citronella essential oil, thyme essential oil and basil essential oil were blended together in different combinations, the activity was also higher than expected. However, a four-component equilibrium mixture was not as effective as quaternary mixtures with one component dominating (Table 1).

The main ingredients of highest antioxidant activity (HAA) are listed in Table 2 (the detailed composition of HAA is included in Table S2). The designed mixture with approximated HAA; EC_50_ 0.65 mg/mL) was composed of: 9.2% of cedarwood essential oil, 54.4% of citronella essential oil, 33.0% of thyme essential oil and 3.4% of basil essential oil. According to the percentage composition, citronellal, thymol, geraniol, citronellol and p-cymene should be leading contributors to antioxidant activity; and indeed, in reality, these compounds showed good antioxidant properties against the DPPH radical cation (EC_50_ of 79.9, 269.0, 24.6 and 80.0 μg/mL for citronellal, thymol, geraniol, citronellol, respectively) [35,36,37,38]. What is more, p-cymene significantly reduced the level of lipid peroxidation and nitrite content in vivo [39]. When citronellal and p-cymene were studied in combinations with different ratio, synergism in antioxidant activity was observed for their binary mixtures (ratio 1 + 1 and 3 + 1, 7 + 1; 15 + 1) against DPPH radical cation [40]. In the HAA mixture designed in this study, the ratio of citronellal and p-cymene was 2.5 and this may have contributed to the high approximated activity that matched well when confirmed experimentally (approximated EC_50_ value of 0.65 mg/mL vs. experimental EC_50_ value of 0.68 mg/mL). The interactions of between other components were not described; however, it is probable that the overall good activity of the HAA is due to additive or synergistic action between constituents.

The mechanism of action of essential oils depends on many factors, as well as their mutual proportions in the mixture. Gutierrez et al. [41] showed that essential oils of thyme and oregano with strong individual antibacterial activity did not show synergistic effects, while binary mixtures of essential oils showing moderate activity in combination with essential oils of marjoram, basil, rosemary or sage resulted in enhanced effects. Mixtures of oregano or thyme essential oil with basil, rosemary or sage essential oil showed additive activity against *Listeria monocytogenes*. [41]. Moreover, higher inhibitory capacity against *Candida albicans*, *Aspergillus niger* and *Staphylococcus aureus* compared to activity of individual essential oils was shown by mixtures of citronella with patchouli and citronella with nutmeg essential oils [42], underlining the complex interactions between ingredients.

Although in recent years there have been more and more works on mutual interactions between the mixture of essential oils using statistical modelling, most often they concern the optimization of the composition of the essential oil mixture in terms of their antimicrobial activity. Bertin et al. [19] optimized a mixture of essential oils obtained from *Plectranthus glandulosus*, *Ocimum gratissimum*, *Cymbopogon citratus*, *Cymbopogon nardus* and *Eucalyptus* spp. The authors prepared 88 essential oil blends that were tested for activity against various strains of microbes. The results allowed the selection of the appropriate composition of the mixture depending on the zones of microbial inhibition. The most significant effects were observed in essential oil mixtures: *P. glandulosus* + *Eucalyptus* spp.) and *P. glandulosus* + *O. gratissimum* [19]. Using the simplex-centroid mixture design methodology Torres-Neto et al. [43] optimized the composition of the mixture of essential oils obtained from oregano, thyme and lemongrass to achieve the best values of minimum inhibitory concentration and minimum bactericidal concentration against *Salmonella enterica* serotype *Enteritidis*, *Escherichia coli* and *Staphylococcus aureus*. An essential oils blend of 75% oregano: 15% thyme: 10% lemongrass showed maximum pathogen inhibition, while an essential oil blend of 50% oregano: 40% thyme: 10% lemongrass showed maximum pathogen inactivation [43]. Research by Ouedrhiri et al. [44] did not show the synergistic effect of a mixture of essential oils from *Myrtus communis*, *Artemisia herba-alba* and *Thymus serpyllum* against *Staphylococcus aureus* and *Escherichia coli*. The single *T. serpyllum* oil was most effective against these strains. However, the use of the simplex-centroid design allowed for the composition of a mixture of essential oils derived from myrtle, wormwood and wild thyme (17.1%, 39.6% and 43.1%, respectively) that showed optimal inhibitory activity against *Bacillus subtillis*. [44].

Planning of the optimization of the mixtures undergoes certain limitations. The sum of all its components must have a constant value ∑x*_i_* = 1 (or 100%), where *i* is the number of ingredients in the mixture (*i* = 1, 2, 3, …, q) [45]. In the current scientific literature relevant to the mixture design, the most commonly described are systems consisting of three components (triangular mixtures). However, there are also works using optimization of mixtures of four components [46]. For this purpose, tetrahedron models are the most commonly used, which are a three-dimensional simplex. To optimize the four-component extraction mixture, Soares et al. [47] used the tetrahedron system; however, all points of the mixtures, in addition to the central one, were on triangular surfaces. The tetrahedron system used by Soares et al. [47] can be described as follows:

a, b, c, d (a = b = c = d = 100%)(1)

ab, ac, ad, bc, bd, cd (a = b = c = d = 50%)(2)

abc0, ab0d, a0cd, 0bcd (a = b = c = d = 33%)(3)

abcd (a = b = c = d = 25%)(4)

**Figure 1:**
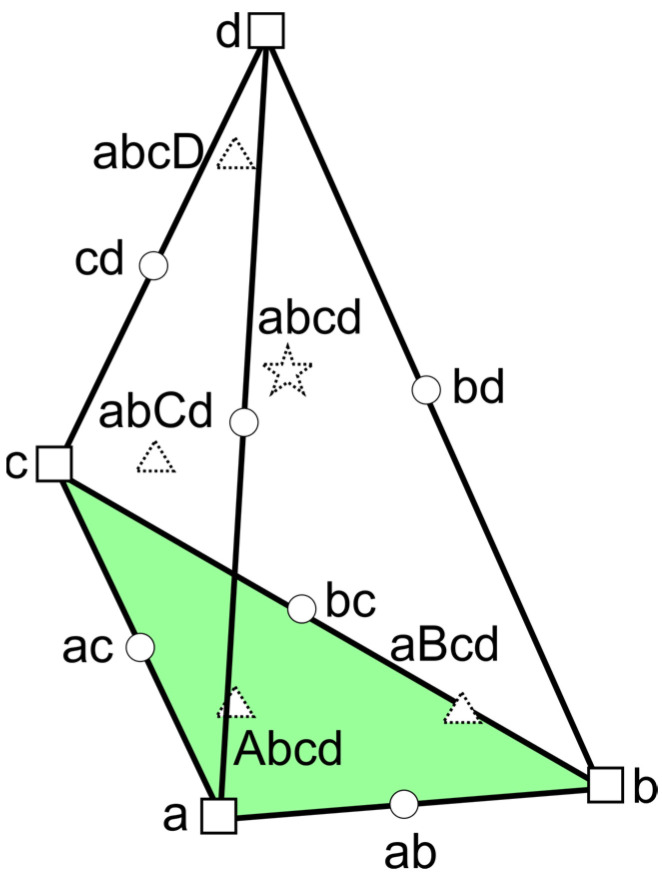
Schematic representation of simplex-lattice mixture design used to optimize essential oil mixtures: cedarwood (a), citronella (b), thyme (c), basil (d). The capital letter indicates the dominant ingredient in the mixture.

As can be seen from the above, the assessment of the interaction between the four components of the mixture occurs only in one point, when components are mixed in equal amounts (4). The scheme of the mixture is shown in Figure 1.

The other experimental model was proposed by Dias et al. [48]. Instead of a three-component mixture (3), the authors introduced a four-component combination (5), which can be presented as follows:

abcD, abCd, aBbd, Abcd (a = b = c = d = 12.5% and A = B = C = D = 62.5%).(5)

The experimental model described by Dias et al. [48] was adapted in this work. A representation of a four-component mixture of the essential oils tested in this study is shown in Figure 1 in a graphic manner.

The first stage of the statistical analysis was ANOVA, an analysis of the results obtained using linear, quadratic and special cubic models. The *p*-value and the coefficient of determination R^2^ adj. were assumed as the acceptance criteria for the model (Table 3). The linear model in the analysed experiment had 3 degrees of freedom. The statistical test of this model showed its significance at the level of F (3,13) = 8.30, *p* < 0.05. In the quadratic model, which was used to analyse the interaction between the individual components, the number of degrees of freedom doubled. This model was statistically significant F (6.7) = 9.5, *p* < 0.05. In this case, the coefficient of determination was also improved. The high level of variation determination indicates a good adjustment of the quadratic model, and 96.25% of the variation was explained by the obtained data (Table 3). Increasing the number of parameters in a special cubic and full cubic model did not show any significant improvement in fit.

In the special cubic model, the number of degrees of freedom was lower than in the quadratic model, and this model was not significant at the assumed level of *p* < 0.5. Due to the peculiarity of the X′X matrix, the cubic model was not analysed. Based on the above results, a quadratic model was chosen for further analysis, which can be recorded with the Equation (6) [15]:

y = *β*_0_ Σ *β_k_* X*_k_* + ΣΣ *β_kj_* X*_k_* X*_j_* + *ε* (6)

where, *k* = 1, …, *q* (with *q* mixture components), *j* = *k*, …, *q*, ε-random factor.

**Figure 2:**
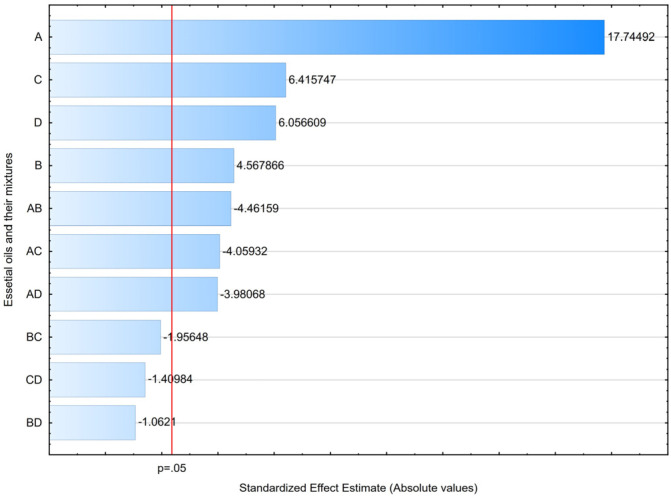
Pareto chart for a quadratic model with the normalized effects of the four experimental factors, in decreasing order of importance (in absolute terms) for the antioxidant activity (EC_50_) response. Essential oil: cedarwood (A), citronella (B), thyme (C), basil (D).

The analysis of the quadratic model’s fit showed statistical significance at the level *p* < 0.001. The lack of fit indicates the accuracy of the selected model (Table 4). The Pareto chart shows the variables that significantly affected the antioxidant activity at the significance level of 95%. Negative values of coefficients for essential oil mixtures (A–D) indicate a beneficial effect on reducing the EC_50_ parameter (Figure 2). The analysis of critical values was carried out to determine the optimal proportions of ingredients in terms of antioxidant properties. The value approximated for the best antioxidant properties was obtained for the mixture with the percentage composition: 9.2 (A):54.4 (B):33.0 (C):3.4 (D). The EC_50_ predicted in the model was 0.65 mg/mL, while experimental data obtained in five replicates for the above composition was EC_50_ of 0.68 mg/mL-confirming a good selection of statistical model.

**Figure 3:**
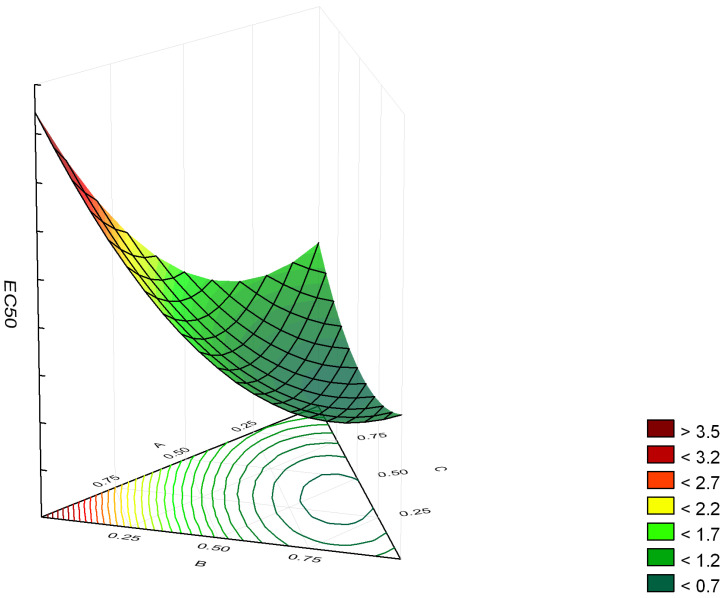
Fitted surface for best mixture OE in terms of antioxidant properties (cedarwood (A), citronella (B), thyme (C), compound D = 0.03)).

In order to represent graphically on the fit surface response graph (Figure 3), the constant value of the component D was assumed for the optimal mixture at the level of 3.3%. The following is the quadratic equation for the optimized values of the adopted model:

EC_50_ = 3.92 × A × 0.97 + 1.01 × B × 0.97 + 1.42 × C × 0.97 − 4.62 × A × 0.97 × B × 0.97 − 4.20 × A × 0.97 × C × 0.97 − 4.12 × 0.03 × A × 0.97 − 2.02 × B × 0.97 × C × 0.97 − 1.10 × 0.03 × B × 0.97 − 1.46 × 0.03 × C × 0.97 + 0.045

where, A, B, C—observations of an independent variable.

## 4. Conclusions

The synergistic properties of natural ingredients are increasingly used. Essential oils show a large variability in composition depending on many external factors, which can affect their mutual interactions. Therefore, in order to optimize their mutual proportions in terms of the highest biological activity, statistical modelling should be considered. Mixture design can be a useful tool to prepare a mixture with optimal properties that can be used to protect food. The presented research shows that by using statistical modelling of Mixture Design it is possible to optimize the composition of a mixture of four essential oils in order to obtain optimal antioxidant activity. More and more often there are scientific papers describing the increased activity of an optimized essential oils mixtures in antimicrobial, food preservation and preparation and other fields [14,46,49,50,51]. These confirm the need for testing for possible interactions between components of the mixture. In the presented work, we revealed that it is possible to approximate the best antioxidant composition of four essential oils using simplex-lattice mixture design and DPPD-tests. Most of the statistical models for mixtures to date are based on ternary designs. The presented studies demonstrated the possibility of predicting antioxidant activity for a four-component mixture of essential oils. The development of the use of the Mixture Design method in industry seems to be the right direction to optimize multi-component systems.

## Figures and Tables

**Table 1:** The experimental and approximated antioxidant activity of essential oils and their mixtures.

Experiment Codes	A (%)	B(%)	C(%)	D(%)		EC_50_	
	Cedarwood	Citronella	Thyme	Basil	Mixtures	Experimental	Approximated
a	100.0	0.0	0.0	0.0	Pure compounds	4.04	3.92
b	0.0	100.0	0.0	0.0		1.08	1.01
c	0.0	0.0	100.0	0.0		1.47	1.42
d	0.0	0.0	0.0	100.0		1.36	1.34
ab	50.0	50.0	0.0	0.0	Binary	1.28	1.31
ac	50.0	0.0	50.0	0.0		1.57	1.62
ad	50.0	0.0	0.0	50.0		1.52	1.60
bc	0.0	50.0	50.0	0.0		0.61	0.71
bd	0.0	50.0	0.0	50.0		0.77	0.90
cd	0.0	0.0	50.0	50.0		0.87	1.01
Abcd	62.5	12.5	12.5	12.5	Quaternary	1.53	1.84
aBcd	12.5	62.5	12.5	12.5		0.60	0.71
abCd	12.5	12.5	62.5	12.5		0.88	0.91
abcD	12.5	12.5	12.5	62.5		1.03	0.94
abcd	25.0	25.0	25.0	25.0	Equilibrium	1.18	0.83
abcd	25.0	25.0	25.0	25.0		0.91	0.83
abcd	25.0	25.0	25.0	25.0		1.01	0.83

**Table 2:** The main constituents of HAA.

Main Constituents *	%
p−Cymene	7.4
Limonene	3.1
γ−Terpinene	2.5
Linalool	2.4
Citronellal	17.0
Methylchavicol	2.7
Citronellol	7.3
Geraniol	11.2
Thymol	14.1
Carvacrol	1.3
Citronellol acetate	1.7
Neil acetate	2.0
β−Elemene	1.3
α−Cedrene	1.6
Thujopsene (Widdrene)	2.1
δ−Cadinene	1.2
Elemol	1.8
Cedrol	1.4

* constituents with percentage in a sum ≥ 1%, HAA—mixture with highest antioxidant activity.

**Table 3:** ANOVA analysis for linear, quadratic and special cubic models.

Model	SS Effect	df Effect	MS Effect	SS Error	df Error	MS Error	F	*p*	R^2^	R^2^ adj.
Linear	6.3696	3	2.1232	3.3237	13	0.2557	8.3044	0.0024	0.6571	0.5780
Quadratic	2.9603	6	0.4934	0.3634	7	0.0519	9.5030	0.0045	0.9625	0.9143
Special cubic	0.2450	4	0.0613	0.1184	3	0.0395	1.5522	0.3740	0.9878	0.9349
Total Adjusted	9.6934	16	0.6058							

**Table 4:** Overall fit of quadratic Model Variances.

	SS	df	MS	F	*p*
Model quadratic	9.3300	9	1.0366	19.9668	0.0003
Total Error	0.3634	7	0.0519		
Lack of Fit	0.3261	5	0.0652	3.4909	0.2375
Pure Error.	0.0374	2	0.0187		
Total Adjusted	9.6934	16	0.6058		

## Data Availability

The data used to support the findings of this study are included.

## Supplementary Materials

The following supporting information can be downloaded at: https://www.mdpi.com/article/10.3390/antiox12030577/s1, Table S1. The chemical composition of used essentials oils. Table S2. The chemical composition of HAA—mixture with highest antioxidant activity.
