# Supplementary material for: Synergistic Antioxidant Activity of Four—Component Mixture of Essential Oils: Basil, Cedarwood, Citronella and Thyme for the Use as Medicinal and Food Ingredient
Source: Antioxidants (Basel). 2023 Feb 25;12(3):577. doi: 10.3390/antiox12030577 (PMC10044885; doi:10.3390/antiox12030577)
Supplement: Supplementary file 1 [file antioxidants-12-00577-s001.zip › antioxidants-2208378-supplementary.pdf]

antioxidants

MDPI

Supplementary

Synergistic Antioxidant Activity of Four–Component Mixture of Essential Oils: Basil, Cedarwood, Citronella and Thyme for the Use as Medicinal and Food Ingredient

Tomasz Baj [1], Grażyna Kowalska [2], Radosław Kowalski [3], Jolanta Szymańska [4], Guoyin Kai [5], Henrique Douglas Melo Coutinho [6], Elwira Sieniawska [7]*

[1] Department of Pharmacognosy with Medicinal Plants Garden, Medical University of Lublin, 1 Chodźki Str., 20-093 Lublin, Poland
[2] Department of Tourism and Recreation, University of Life Sciences in Lublin, 15 Akademicka Str., 20-950 Lublin, Poland
[3] Department of Analysis and Food Quality Assessment, University of Life Sciences in Lublin, 8 Skromna Str., 20-704 Lublin, Poland
[4] Department of Integrated Paediatric Dentistry, Chair of Integrated Dentistry, Medical University of Lublin, 6 Chodźki Str., 20-093 Lublin, Poland
[5] Zhejiang Provincial International S&T Cooperation Base for Active Ingredients of Medicinal and Edible Plants and Health, Jinhua Academy, School of Pharmaceutical Sciences, Zhejiang Chinese Medical University, Hangzhou, Zhejiang, 310053, China
[6] Departamento de Química Biológica, Universidade Regional do Cariri, Rua Cel. Antônio Luíz - Pimenta, Crato - CE, 63105-110, Brazil
[7] Department of Natural Products Chemistry, Medical University of Lublin, 1 Chodźki Str., 20-093 Lublin, Poland
* Correspondence: esieniawska@pharmacognosy.org

Citation: Baj, T.; Kowalska, G.; Kowalski, R.; Szymańska, J.; Kai, G.; Coutinho, H.D.M.; Sieniawska, E. Synergistic Antioxidant Activity of Four—Component Mixture of Essential Oils: Basil, Cedarwood, Citronella and Thyme for the Use as Medicinal and Food Ingredient. *Antioxidants* **2023**, *12*, 577. https://doi.org/10.3390/antiox12030577

Academic Editor: Stanley Omaye

Received: 24 January 2023
Revised: 13 February 2023
Accepted: 15 February 2023
Published: 22 February 2023

Copyright: © 2023 by the authors. Submitted for possible open access publication under the terms and conditions of the Creative Commons Attribution (CC BY) license (https://creativecommons.org/licenses/by/4.0/).

Table S1. The chemical composition of used essentials oils.

RI*	Compounds	Area (%)			
		Citronella	Basil	Cedarwood	Thyme
927	α–Thujene				0.99
935	α–Pinene		0.18		1.39
952	Camphene				0.80
974	sabinene		0.11	0.11	
980	β–Pinene		0.25		0.19
990	β–Myrcene	0.07	0.14		1.56
1005	Fenchene				0.06
1008	α–Phellandrene				0.05
1019	α–Terpinene				1.96
1028	p–Cymene		0.55		22.42
1032	Limonene	5.26	0.53		0.75
1035	1,8–Cineole		4.51		0.88
1048	β–Ocimene		0.69		
1061	γ–Terpinene		0.77		7.35
1075	cis-Sabinene hydrate				0.17
1088	Terpinolene				0.25
1103	Linalool	0.64	1.44		6.20
1153	Camphor		0.30		0.21
1155	Citronellal	31.23			
1180	Borneol				1.65
1187	Terpinen–4–ol		0.23		0.96
1196	Isopulegol	0.52			
1202	α–Terpineol				1.27
1203	Methyl chavicol		80.63		
1230	Citronellol	13.42			
1221	Fenchyl acetate		0.13		
1241	Neral	1.18			
1242	Thymol methyl ether			0.85	0.40

1249	Linalyl acetate				0.15
1254	Geraniol	20.55			0.10
1270	Geranial	1.67			
1288	Bornyl acetate		0.12		
1291	Carvacrol acetate				0.06
1301	Thymol				42.87
1310	Carvacrol				4.08
1348	Citronellol acetate	3.05			
1350	α–Terpineol acetate			0.06	
1358	Eugenol	0.73	0.47		
1376	Geranyl acetate				0.16
1377	Neil acetate	3.65			
1391	α–Copaene			0.49	0.06
1395	β–Elemene	2.15	0.23	1.18	
1401	Isoeugenol methyl ether		0.40		
1404	Isolongifolene			0.44	
1414	α–Longipinene			1.11	
1421	Longifolene			0.35	
1428	α–Cedrene			17.36	
1429	β–Caryophyllene		0.52		2.53
1436	β–Cedrene			5.69	
1438	α–Bergamotene		2.11		
1447	(+)–Thujopsene (Widdrene)			23.21	
1453	Cis-β–Farnesene			0.30	
1457	β–Sesquiphellandrene			0.11	
1466	α–Humulene				0.15
1474	Allo–Aromadendrene			1.13	
1479	β–Barbatene			0.90	
1485	Selina–4,11–diene			1.32	
1490	Isocaryophyllene		0.23		
1491	Germacrene D	1.20			
1493	β–chamigrene			2.01	

1499	α–Guaiene			0.35	
1503	α–Chamigrene			0.47	
1505	α–Cadinene	0.63			
1506	α–Selinene			0.34	
1514	β–Himachalene			4.70	
1519	(+)-Cuparene			8.14	
1522	γ– Cadinene	0.50	0.36		
1525	δ–Cadinene	2.12		0.81	0.05
1531	γ–Elemene			1.66	
1545	ar-curcumene			2.12	
1558	Elemol	3.25			
1595	Caryophyllene oxide				0.26
1613	Cedrenol			1.26	
1622	Cedrol			15.33	
1627	Widdrol			1.46	
1632	Curlone			0.90	
1641	Epicedrol			0.37	
1643	γ–Muurolene	0.50	0.56		
1647	α–acorenol	0.31			
1655	τ–Cadinol	0.74			
1668	α– Cadinol	0.57			
1670	α–Eudesmol	0.53			
1672	β–Humulene			0.40	

RI—retention indices measured on capillary column ZB-5

Table S2. The chemical composition of HAA – mixture with highest antioxidant activity.

Constituents of HAA	%
α–Thujene	0.3
α–Pinene	0.5
Camphene	0.3
β–Pinene	0.1
β–Myrcene	0.6
α–Terpinene	0.6
p-Cymene	7.4
Limonene	3.1
1,8-Cineole	0.4
γ–Terpinene	2.5
cis-Sabinene hydrate	0.1
Terpinolene	0.1
Linalool	2.4
Camphor	0.1
Citronellal	17.0
Borneol	0.5
Terpinen–4–ol	0.3
Isopulegol	0.3
α–Terpineol	0.4
Methyl chavicol	2.7
Citronellol	7.3
Neral	0.6
Thymol methyl ether	0.2
Linalyl acetate	0.0
Geraniol	11.2
Geranial	0.9
Thymol	14.1
Carvacrol	1.3
Citronellol acetate	1.7
Eugenol	0.4
Geranyl acetate	0.1
Neil acetate	2.0
α–Copaene	0.1
β–Elemene	1.3
α–Longipinene	0.1
α–Cedrene	1.6
β–Caryophyllene	0.9

β–Cedrene	0.5
α–Bergamotene	0.1
(+)-Thujopsene (Widdrene)	2.1
Allo–Aromadendrene	0.1
β–Barbatene	0.1
Selina–4,11-dien	0.1
Germacrene D	0.7
β–chamigrene	0.2
α–Cadinene	0.3
β–Himachalene	0.4
(+)–Cuparene	0.7
γ–Cadinene	0.3
δ-Cadinene	1.2
γ–Elemene	0.2
ar–Curcumene	0.2
Elemol	1.8
Caryophyllene oxide	0.1
Cedrenol	0.1
Cedrol	1.4
Widdrol	0.1
Curlone	0.1
γ–Muurolene	0.3
α–Acorenol	0.2
τ–Cadinol	0.4
α Cadinol	0.3
α–Eudesmol	0.3
